# Effekte der Qualitätssicherung in der Endoprothetik: Eine Evaluierung der Entwicklung der halbjährlichen Klinikauswertungen des Endoprothesenregister Deutschland (EPRD)

**DOI:** 10.1007/s00132-025-04639-2

**Published:** 2025-04-01

**Authors:** Katrin Osmanski-Zenk, Wolfram Mittelmeier, Oliver Melsheimer

**Affiliations:** 1 Orthopädische Klinik und Poliklinik, Universitätsmedizin Rostock, Doberaner Straße 142, 18057 Rostock, Deutschland; 2 EPRD Deutsche Endoprothesenregister gGmbH, Berlin, Deutschland Straße des 17. Juni 106–108 , 10623

**Keywords:** EndoCert, Zertifizierung, Versorgungsqualität, Gelenkersatz, Wechselverhältnis, EndoCert, Certification, Quality of care, Joint replacement, Revisions rate

## Abstract

**Hintergrund:**

Die Qualitätssicherung in der Endoprothetik basiert auf etablierten Systemen wie dem Endoprothesenregister Deutschland (EPRD) und dem EndoCert-Zertifizierungssystem. Diese Systeme ermöglichen durch gezielte Rückmeldungen und Audits eine kontinuierliche Verbesserung der Versorgungsqualität. Nach Einführung der halbjährlichen EPRD-Klinikauswertungen wurden für die Kliniken detaillierte Analysen und Vergleiche ihrer Wechselverhältnisse bei Hüft- und Knieendoprothesen (HTEP, KTEP) möglich.

**Ziel der Arbeit:**

Ziel dieser Studie war es, die standardisierten Wechselverhältnisse (SWV) der EPRD-Kliniken zu vergleichen und zu bewerten.

**Material und Methoden:**

Untersucht wurde, ob auffällig schlechte Ergebnisse in früheren Auswertungen zu einer Verbesserung über die Zeit führten. Die Analyse basierte auf Klinikauswertungen von Juni 2020 und Dezember 2023 für elektive HTEP mit zementfreiem Schaft sowie Standard-KTEP. Von den 603 Kliniken mit HTEP-Rückmeldungen wurden 315 betrachtet, bei den 588 KTEP-Kliniken waren es 360.

**Ergebnisse:**

Die Ergebnisse zeigen, dass regelmäßige Feedbackmechanismen die Versorgungsqualität verbessern können. So konnten 69,2 % der Kliniken ihre Qualität bei HTEP-Versorgungen halten oder verbessern, bei KTEP waren es sogar 70,6 %. Es wurde keine Abhängigkeit von Fallzahlen festgestellt, was darauf hinweist, dass alle EPRD-Kliniken gleichermaßen von den Rückmeldungen profitieren können.

**Diskussion:**

Die Klinikauswertungen ermöglichen eine präzise Nachverfolgung der Ergebnisse, fördern Transparenz und legen Verbesserungspotenziale offen. Angesichts gesetzlicher Neuerungen wie dem Implantateregistergesetz und der Krankenhausreform ist es essenziell, bestehende Systeme wie das EPRD und EndoCert weiter zu stärken und noch stärker in die klinische Praxis zu integrieren, um die Versorgungsqualität nachhaltig zu sichern.

**Graphic abstract:**

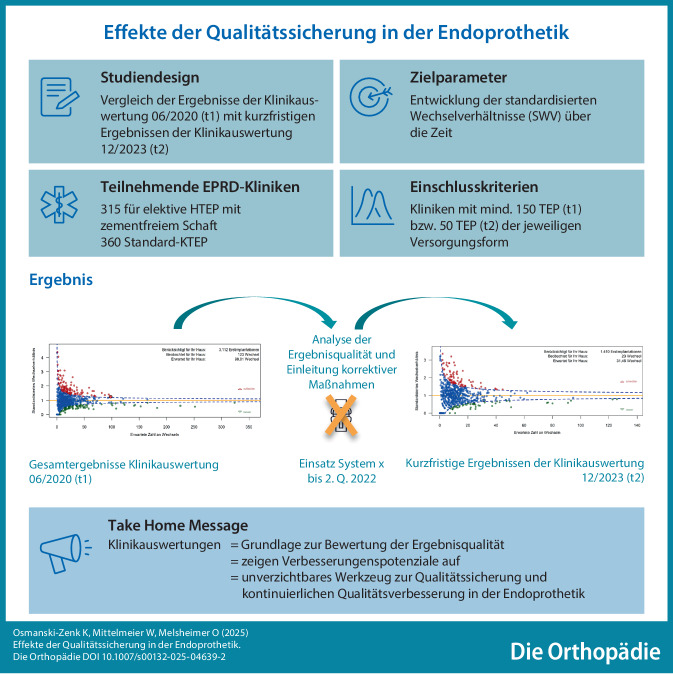

## Abstract (en)

**Background:**

Quality assurance in arthroplasty is based on established systems such as the German Arthroplasty Registry (EPRD) and the EndoCert certification system. These systems enable continuous improvement in the quality of care through targeted feedback and audits. After the introduction of the semi-annual EPRD hospital evaluations, detailed analyses and comparisons of their revision rates for hip and knee arthroplasties (THA, TKA) became possible for the hospitals.

**Aim of the study:**

The aim of this study was to compare and evaluate the standardised revision ratios (SRR) of the EPRD clinics.

**Materials and methods:**

The study analysed whether poor results in previous evaluations led to an improvement over time. The analysis was based on hospital evaluations from June 2020 and December 2023 for elective THA with cementless stems and standard TKA. Of the 603 clinics with THA responses, 315 were analysed, compared to 360 of the 588 TKA clinics.

**Results:**

The results show that regular feedback mechanisms can improve the quality of care; 69.2% of clinics were able to maintain or improve the quality of their THA care and 70.6% of clinics were able to maintain or improve their TKA care. No dependence on case volumes was found, which indicates that all EPRD clinics can benefit equally from the feedback.

**Discussion:**

The hospital evaluations enable precise tracking of results, promote transparency and reveal potential for improvement. In view of new legislation such as the Implant Register Act and the hospital reform, it is essential to further strengthen existing systems such as the EPRD and EndoCert and integrate them even more closely into clinical practice in order to ensure the quality of care in the long term.

## Hintergrund und Fragestellung

Die Deutsche Krankenhausgesellschaft (DKG) hat in ihrem aktuellen Positionspapier vom März 2024 ein neues System zur Qualitätssicherung und -verbesserung der Patientenversorgung vorgeschlagen, um die ineffizienten und bürokratischen Strukturen der bisherigen gesetzlichen Qualitätssicherung (QS) zu reformieren. Ziel ist eine ganzheitliche Betrachtung der Versorgungsqualität, die nicht nur die Erfüllung einzelner Anforderungen, sondern die Gesamtheit der Patientenversorgung in den Fokus rückt. Die DKG fordert spezifische Qualitätsanforderungen für medizinische Behandlungen und Verfahren zur Qualitätsbeurteilung [2]. Geraedts und de Cruppé [4] kommen ebenfalls in ihrer Analyse zu möglichen Effekten der gesetzlichen Qualitätssicherung, auf Basis nationaler und internationaler Literatur, zu dem Ergebnis, dass die Effekte auf die Versorgungsqualität überwiegend schwach sind, was auf Mängel in der Gestaltung und Validität der Berichte hinweist, sodass eine Überarbeitung der gesetzlichen QS notwendig erscheint.

Das von der DKG geforderte neue Qualitätssicherungssystem (QSS), welches durch Audits und Maßnahmen zur Verbesserung der Qualität unterstützen und gleichzeitig primär die Ursachen von Qualitätsdefiziten analysieren und beseitigen soll, ist dementsprechend in der Endoprothetik bereits umfassend implementiert. Die bestehenden QSS der EndoCert GmbH und des Endoprothesenregister Deutschland (EPRD) bieten bereits seit über 12 Jahren alle notwendigen Grundlagen für eine effektive Qualitätsbewertung und -sicherung in diesem Bereich [6, 7].

Mit der Einführung halbjährlicher Klinikauswertungen durch das EPRD im Jahr 2018 wurde ein wichtiger Schritt zur kontinuierlichen Verbesserung der Versorgungsqualität unternommen. Diese Auswertungen ermöglichen es Kliniken, u. a. ihre Wechselverhältnisse bei Hüft- und Knieversorgungen systematisch zu analysieren und zu vergleichen [3].

Ziel dieser Analyse ist es, die standardisierten Wechselverhältnisse der am EPRD teilnehmenden Kliniken seit der Implementierung der Klinikauswertungen zu vergleichen und zu bewerten. Die Ergebnisse sollen nicht nur Verbesserungspotenziale im Rahmen der Weiterentwicklung der Qualitätssicherungssysteme aufzeigen, sondern auch dazu beitragen, den Nutzen der Klinikauswertungen zu maximieren, um die Patientenversorgung im Bereich der Endoprothetik nachhaltig zu optimieren. Es soll die Hypothese geprüft werden, ob auffallend schlechte Ergebnisse, die sich in der Klinikauswertung zeigten, zur Reduktion der standardisierten Wechselverhältnisse über die Zeit führten.

Darüber hinaus sind die Erkenntnisse aus den Klinikauswertungen nicht nur für die Kliniken selbst von Bedeutung, sondern auch für alle Interessengruppen im Gesundheitswesen, die an einer kontinuierlichen Verbesserung der Versorgungsqualität interessiert sind. Speziell vor dem Hintergrund der gesetzlich festgelegten Einführung des Implantateregister Deutschland (IRD) [1], welches u. a. die Erfassung der Endoprothesen beabsichtigt, soll die Wichtigkeit und das Potenzial des EPRD und das damit verbundene Berichtswesen kritisch geprüft werden, da es nicht nur zur Verbesserung der Versorgungsqualität beiträgt, sondern auch als Grundlage für evidenzbasierte Entscheidungen in der klinischen Praxis dient.

## Material und Methodik

Diese Auswertung basiert auf einer Gegenüberstellung der Ergebnisse der Klinikauswertung, die den Kliniken im Juni 2020 zur Verfügung gestellt wurde, und der kurzfristigen Ergebnisse der Klinikauswertung aus dem Dezember 2023. In den Klinikauswertungen wird für jede Versorgungsform die für eine Klinik beobachtete Zahl der in der Nachverfolgungszeit bereits gewechselten Primärversorgungen mit dem dafür erwarteten Wert verglichen. Dieser erwartete Wert wird wie beim Log-Rang-Test anhand der Gesamtdaten berechnet [20].

In den jeweiligen Klinikauswertungen werden die beobachtete und die erwartete Wechselzahl in Relation gesetzt und grafisch in Form von „funnel plots“ dargestellt [10]. Der Quotient aus beobachteter und erwarteter Wechselzahl wird als standardisiertes Wechselverhältnis (SWV) bezeichnet. Liegt das SWV unter 1, sind in dieser Klinik entsprechend weniger Wechsel tatsächlich beobachtet worden als erwartet worden wären; liegt es über 1, so gab es mehr Wechsel als erwartet. Ist das SWV für eine Klinik zum 2,5-%-Niveau signifikant größer als 1, so wird der Punkt dieser Klinik im „funnel plot“ rot eingefärbt; ist das SWV zum 2,5-%-Niveau signifikant kleiner als 1, so wird er grün gezeichnet.

Die Auswertungen aus dem Juni 2020 berücksichtigten alle im EPRD dokumentierten Eingriffe in Nachverfolgung bis zum 30.09.2019. In die kurzfristige Ergebnisdarstellung der Klinikauswertung 2023‑2 aus dem Dezember 2023 flossen dagegen nur die Eingriffe aus dem Zeitraum vom 01.10.2020 bis zum 31.03.2023 ein, d. h. der hierfür betrachtete Zeitraum überlappte sich nicht mit dem der vorgenannten Auswertungen.

Die standardisierten Wechselverhältnisse der erstgenannten Auswertungen wurden für die verbreitetsten Versorgungsformen, d. h. die elektiven Hüfttotalendoprothesen(HTEP)-Versorgungen mit zementfreiem Schaft und die Standard-Knietotalendoprothesen(KTEP)-Versorgungen, mit denen der letztgenannten deskriptiv verglichen, um die Ergebnisentwicklung aufzuzeigen. Da die SWV-Werte bei niedrigen Fallzahlen sehr stark schwanken können, wurden hierfür ausschließlich die Ergebnisse der Kliniken betrachtet, für die sich für den Betrachtungszeitraum bis zum 30.09.2019 mindestens 150 Versorgungen für die jeweilige Versorgungsform in Nachverfolgung befanden und für den Zweieinhalbjahreszeitraum vom 01.10.2020 bis zum 31.03.2023 mindestens 50. Von den 603 Kliniken, die mit den Klinikauswertungen 2020‑1 und 2023‑2 Rückmeldung über ihre elektiven HTEP-Versorgungen mit zementfreiem Schaft erhalten hatten, wurden daher 315 betrachtet; von den 588 Kliniken mit Rückmeldungen über ihre Standard-KTEP-Versorgungen 360.

## Ergebnisse

### Allgemeine Entwicklung der Klinikauswertung HTEP-Versorgung

**Abb. 1:**
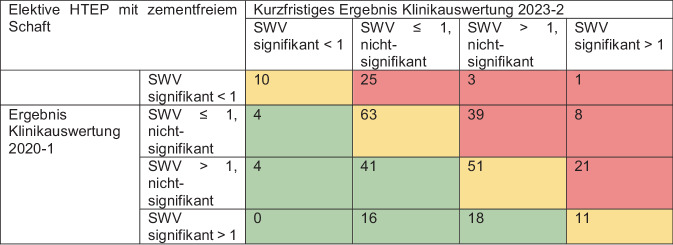
Entwicklung der Standardisierten Wechselverhältnisse (SWV) nach Hüftendoprothesen-Versorgung (HTEP) auf Klinikebene

Im Bereich der elektiven HTEP-Versorgungen mit zementfreiem Schaft lag bei 162 der 315 betrachteten Kliniken bei der Klinikauswertung 2020‑1 ein standardisiertes Wechselverhältnis von über 1 vor, d. h. für sie wurden mehr Wechsel beobachtet als erwartet wurde. Bei 45 davon lag das Wechselverhältnis dabei sogar im roten Bereich, d. h. es war zum 2,5-%-Niveau signifikant erhöht. 16 dieser 45 Kliniken lagen bei den kurzfristigen Ergebnisdarstellung in der Klinikauswertung 2023‑2 nicht nur nicht im roten Bereich, sondern ihr Wechselverhältnis lag nun bei weniger als 1. Bei insgesamt 101 der 162 Kliniken mit Wechselverhältnis über 1 war dies auch noch bei den kurzfristigen Ergebnissen in Auswertung 2023‑2 der Fall (Abb. 1). Auffallend ist ein Fall, in dem eine zunächst erfolgreiche Klinik, die in der Klinikauswertung 2020‑1 im grünen Bereich lag, nahezu vollständig von ihren bewährten Implantatsystemen abrückte und auf alternative HTEP-Systeme umstellte. Diese Entscheidung führte zu einem signifikanten Anstieg der Revisionsereignisse. In der Folge platzierte sich die Klinik in den kurzfristigen Ergebnissen der Klinikauswertung 2023‑2 im auffällig negativen Bereich des „funnel plots“.

### Allgemeine Entwicklung der Klinikauswertung Knie-TEP-Versorgung

**Abb. 2:**
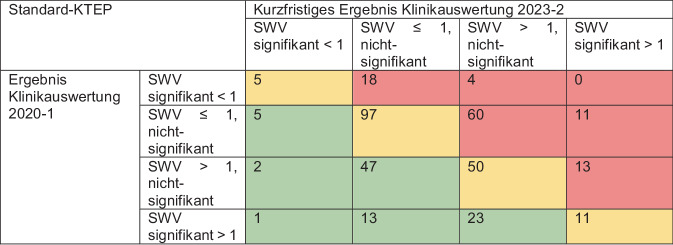
Entwicklung der Standardisierten Wechselverhältnisse (SWV) nach Knieendoprothesen-Versorgung (KTEP) auf Klinikebene

Bei den Standard-KTEP-Versorgungen lag für 160 von 360 betrachteten Kliniken das standardisierte Wechselverhältnis bei der Auswertung 2020‑1 bei über 1. Bei 97 dieser 160 Kliniken war dies auch bei der kurzfristigen Ergebnisdarstellung in der Auswertung 2023‑2 noch der Fall, 63 hatten sich aber auf ein SWV von unter 1 verbessert. Drei Kliniken konnten sich dabei sogar in den grünen Bereich verbessern. Besonders bemerkenswert ist dabei der Fall einer Klinik, die in der Auswertung 2020‑1 noch signifikant mehr Wechsel als erwartet ausgewiesen bekommen hat, bei der kurzfristigen Ergebnisdarstellung in der Auswertung 2023‑2 nun aber signifikant weniger (Abb. 2).

### Fallbeispiel Entwicklung der Klinikauswertung

Die Klinik, die sich innerhalb des Betrachtungszeitraums von signifikant erhöhten Wechselverhältnissen auf signifikant niedrigen SWV verbesserte, erreichte diese Qualitätsverbesserung unter Reduktion und letztendlich der Einstellung des Einsatzes eines bestimmten Implantatsystems.

Die Klinikauswertung 2020‑1 zeigte mit insgesamt 139 KTEP-Versorgungen eines bestimmten Systems einen SWV von 3,02 (Tab. 1). Anhand der kurzfristigen Ergebnisse der Klinikauswertung 2023‑2 wurde sichtbar, dass dieses Implantatsystem nur noch bis zum 2. Quartal 2022 eingesetzt wurde. Insgesamt wurde eine deutliche Ergebnisverbesserung auch mit den bestehenden Implantatsystemen erreicht (Tab. 2).

**Tab. 1:** Ergebnisse der verwendeten Implantatsysteme aus der Klinikauswertung 2020-1 der Beispielklinik

Knie-TEP 2020‑1			Wechseloperationen	
*System Femurkomponente*	*System Tibiaträger*	*Anzahl*	*Beobachtet*	*Erwartet*
System 1, Femur A	System 1, Tibia A	1151	36	31,43
System 1, Femur B	System 1, Tibia B	332	11	9,02
System 1, Femur A	System 1, Tibia C	139	12	3,98
System 2	System 2	26	0	1,46
System 3	System 3	20	0	0,36

**Tab. 2:** Kurzfristige Ergebnisse der verwendeten Implantatsysteme aus der Klinikauswertung 2023-2 der Beispielklinik

Knie-TEP 2023‑2			Wechseloperationen	
*System Femurkomponente*	*System Tibiaträger*	*Anzahl*	*Beobachtet*	*Erwartet*
System 1, Femur A	System 1, Tibia A	484	3	8,00
System 1, Femur B	System 1, Tibia B	205	1	3,37
System 4	System 5	26	0	0,50
System 5	System 5	14	0	0,50
System 1, Femur A	System 1, Tibia C	12	0	0,40
Andere Kombinationen		19	0	0,40

### Entwicklung der Klinikauswertung in Abhängigkeit von den Klinikfallzahlen

Unsere Ergebnisse zeigten keine Fallzahlabhängigkeit hinsichtlich der Ergebnisentwicklung in den hier verglichenen Klinikauswertungen.

## Diskussion

Ein wichtiger Aspekt der kontinuierlichen Verbesserung der Versorgungsqualität ist die konsequente Analyse und Bewertung der eignen Ergebnisqualität, die aus Qualitätsberichten wie der Klinikauswertung des EPRD abgeleitet werden können. Unsere Ergebnisse zeigen, dass durch regelmäßige Evaluierungen und Feedbackmechanismen die Versorgungsqualität, gemessen anhand der Wechselereignisse, deutlich verbessert werden kann.

Mit der Bereitstellung der Klinikauswertungen, die die teilnehmenden Kliniken erstmals im Juni 2018 erhielten, hat sich das Berichtswesen in der Qualitätssicherung der Endoprothetik aus der Sicht der Autoren grundlegend verändert. Kliniken können auf umfassendere und differenzierte Daten weit über den stationären Aufenthalt hinaus zugreifen. Berichte anderer QSS, wie die externe Qualitätssicherung oder das QSR-Verfahren der AOK, boten nur einen begrenzten Blick auf bestimmte Patientengruppen oder die frühen postoperativen Komplikationen während des stationären Aufenthalts. Diese Einschränkungen behinderten eine umfassende Analyse und die Entwicklung gezielter Maßnahmen zur Qualitätsverbesserung [4]. Limitierend ist jedoch zu erwähnen, dass in der EPRD-Klinikauswertung die Nachverfolgbarkeit der Patienten sich auf die vdek- und AOK-Versicherten beschränkt. Obwohl diese beiden Verbände etwa 75 % der gesetzlich Versicherten in Deutschland repräsentieren, bleibt noch ein Viertel von Patienten unberücksichtigt [18]. Bei gleichen Therapieverfahren ist eine fundierte Aussage dennoch ermöglicht anhand der vorliegenden Zahlen.

Die Einführung der Klinikauswertung bewirkte eine grundlegende Neuausrichtung des Berichtswesens, indem diese eine detaillierte und kontinuierliche Nachverfolgung der Ergebnisse von endoprothetischen Eingriffen bezüglich Implantaten und Kliniken ermöglicht hat. Kliniken erhalten nun individuelle Auswertungsberichte, die ihnen helfen, ihre eigenen Ergebnisse im Kontext nationaler Daten zu bewerten und gezielte Maßnahmen zur Verbesserung der Versorgungsqualität einzuleiten. In einer vorherigen Veröffentlichung unserer Arbeitsgruppe, die Empfehlungen zur Interpretation der verschiedenen EPRD-Berichte aussprach, wurde bereits verdeutlicht, inwiefern die Erkenntnisse zu Anpassungen der Klinikstrukturen und -prozesse genutzt werden müssen, um positive Effekte in der Ergebnisqualität sicherzustellen [14].

Diese umfassende Datenlage fördert nicht nur die Transparenz, sondern auch die Möglichkeit zur Identifikation von Verbesserungspotenzialen in der Patientenversorgung. Der Zugang zu detaillierten Informationen über Implantatstandzeiten und Wechselereignissen ermöglicht es den Kliniken, fundierte Entscheidungen zu treffen und ihre Behandlungsstrategien entsprechend anzupassen.

Bei der Interpretation unserer Ergebnisse ist zu berücksichtigen, dass wir eine Methodik gewählt haben, nach welcher sich die Betrachtungszeiträume für den Vergleich nicht überlappen. Da das Verfahren der standardisierten Wechselverhältnisse als selbstzentrierendes System betrachtet werden muss, führt eine signifikante Qualitätsverbesserung in einigen Kliniken statistisch zwangsläufig zu einer relativen Verschlechterung in anderen Kliniken – auch wenn dabei objektiv keine tatsächliche Verschlechterung vorliegt. Demzufolge kann auf Basis der betrachteten Daten keine grundsätzliche Verschiebung des Qualitätsniveaus in die eine oder andere Richtung nachgewiesen werden. Im aktuellen EPRD-Jahresbericht, der die jahresbezogene Entwicklung der Standzeiten analysiert, wird für die zementfreie HTEP-Versorgung ein gleichbleibend hohes Qualitätsniveau und für die KTEP-Versorgung eine sogar verbesserte Versorgungsqualität festgestellt [5]. Einerseits bedeutet das, dass Kliniken die nachweislich eine Qualitätsverbesserung vor allem in der KTEP-Versorgung über die Zeit erreicht haben, dies durch gezielte und aktive Maßnahmen in ihrer Struktur oder ihren Prozessen oder durch Schulungsmaßnahmen erzielen konnten. Andererseits ist bereits die Sicherstellung eines konstanten SWV als Erfolg zu verstehen, da sich das allgemeine Qualitätsniveau verbessert hat. Nach diesem Verständnis zeigen unsere Ergebnisse, dass 69,2 % der Kliniken bei HTEP-Versorgungen ihr Qualitätsniveau verbessert bzw. konstant halten konnten, bei KTEP-Versorgungen sogar 70,6 %. Ebenso konnten wir keine Fallzahlabhängigkeit bei den Kliniken nachweisen, die sich im Laufe der Zeit verbesserten. Dies legt nahe, dass alle am EPRD teilnehmenden Kliniken die gleichen Möglichkeiten zur Qualitätssteigerung haben. Unsere Arbeitsgruppe konnte in einer früheren Untersuchung innerhalb der EndoCert-Kliniken bereits zeigen, dass kein Zusammenhang zwischen der 3‑Jahres-Revisionsrate und der Versorgungshäufigkeit innerhalb zertifizierter Einrichtungen besteht, unabhängig davon, ob es sich um ein EPZ oder ein EPZmax handelt [13]. Ähnlich zeigt sich bei den EPRD-Kliniken, dass Kliniken, die qualitätssichernde Strukturen und Prozesse etablieren und diese durch Analysen ihrer Qualitätsberichte – einschließlich des EPRD – konsequent nutzen, eine kontinuierliche Verbesserung der Versorgungsqualität erreichen können. Trotz des erheblichen wirtschaftlichen Drucks auf die Kliniken und der gleichzeitigen Reduzierung der Personaldichte [8, 9], konnte die Versorgungsqualität insgesamt auf einem guten Niveau gehalten werden, was darauf hindeutet, dass die implementierten Qualitätssicherungssysteme effektiv zur Stabilität und Verbesserung der Patientenversorgung beitragen. Inwiefern das Berichtswesen des EPRD dazu beigegesteuert hat, die eigenen Strukturen und Prozesse zu hinterfragen und anzupassen, kann an dieser Stelle nicht beantwortet werden. Unser positives Klinikbeispiel jedoch verdeutlicht das Potenzial dieser Berichte und die damit im Zusammenhang stehenden datenbasierten Auswirkungen auf die Versorgungsqualität. Der Beispielklinik ist in kurzer Zeit durch das Absetzen eines einzigen Implantatsystems gelungen, das SWV von signifikant erhöhten auf signifikant niedrigen Ergebnissen über alle KTEP-Versorgungen hinweg zu reduzieren. Es sind ebenso Fälle bekannt, die im Verlauf die SWV-Werte positiv beeinflussen konnten, indem beispielsweise von der Versorgung mit unikondylären Knieendoprothesen, aufgrund erhöhter Revisionsraten, abgesehen wurde. Straub et al. [17] zeigten deutlich höhere Revisionswahrscheinlichkeiten nach Unischlitten-Versorgung im Vergleich zu Standard-KTEP-Versorgungen. Somit können die Erkenntnisse der Klinikauswertungen dazu führen, entweder durch das Absetzen eines bestimmten Systems oder sogar einer ganzen Versorgungsform, die Versorgungsqualität zu steigern. Wenn jedoch ein Systemwechsel zur Verschlechterung der klinikeigenen SWV-Werte führt, wie die Beispielklinik in unseren Ergebnissen nach HTEP-Versorgung, sollte die Klinik nach dieser Information erneut gegensteuern und die Auswirkungen der korrektiven Maßnahmen in der nächsten Klinikauswertung unter Berücksichtigung der kurzfristigen Entwicklungen überprüfen. Wie das zuletzt genannte Beispiel verdeutlicht, darf ein Implantatsystemwechsel, welches mit einer Lernkurve [11, 16] einhergeht, nicht zu drastischen Qualitätsverlusten führen. Nicht bekannt ist bei diesem Beispiel, was der Entscheidung zum Systemwechsel zugrunde lag, sei es durch den Wechsel des Chefarztes, des Operateurteams oder wegen monetärer Ursachen.

Das EPRD, als freiwilliges Register, stellt die klinikbezogenen Revisionsraten nicht der Öffentlichkeit zur Verfügung. Das schwedische Endoprothesenregister hingegen, welches gesetzlich verpflichtend ist, veröffentlicht die Ergebnisse aller Kliniken in den Jahresberichten und erhöht somit die Transparenz für die Interessensgruppen und zeitglich den Druck auf die Klinik zur Verbesserung ihrer Versorgungsqualität [19]. Maximiert wird die Transparenz im britischen Endoprothesenregister (NJR), welches eine Homepage mit sowohl klinik- als auch operateursbezogenen Ergebnissen, wie z. B. der 90-Tage-Sterblichkeit und „patient reported outcome measure“, bereithält [12]. Dieser Zugang ermöglich den Patienten eine ergebnisbasierte Entscheidungsfindung, nicht nur für eine bestimmte Klinik, sondern auch für einen Operateur und fördert somit Transparenz, Vertrauen und die Auswahl der bestmöglichen Versorgung auf Basis objektiver Qualitätsindikatoren. Ergänzend dazu haben die Operateure Zugriff auf detaillierte Auswertungen ihrer eigenen Ergebnisse und die Möglichkeit für ein Benchmark zur Förderung der eignen Ergebnisqualität [15].

## Schlussfolgerung

Insgesamt lässt sich festhalten, dass Qualitätssicherungssysteme nicht nur zur Steigerung der Transparenz beitragen, sondern auch einen direkten Einfluss auf die Verbesserung der Versorgungsstandards ausüben können.

Durch das Implantateregistergesetz (IRegG) sowie die NRW-Krankenhausreform werden die erfolgreich etablierten Qualitätssicherungssysteme EndoCert und EPRD infrage gestellt. Die Vernetzung von risikoadjustierten Registerdaten und ergebnisorientierten Audits vor Ort ist international richtungsweisend. Trotz ihrer entscheidenden Rolle in der letzten Dekade, in der sie maßgeblich zur Weiterentwicklung der Qualitätssicherung in der Endoprothetik beigetragen haben, stehen sie nun vor der Herausforderung, sich gegen nicht validierte evidenzbasierte Qualitätsstandards zu behaupten. Zudem wird ein Implantateregister angestrebt, das bislang jedoch noch keine umfassende Produktdatenbank etabliert hat und auf die Erfahrung des EPRD verzichtet. Diese Entwicklungen werfen Fragen hinsichtlich der Validität und Verlässlichkeit der zukünftigen Qualitätsstandards auf. Angesichts der gesetzlichen Anforderungen zur Qualitätssicherung ist es von zentraler Bedeutung, die bewährten Verfahren der bestehenden Systeme zu erhalten und deren Integration in zukünftige gesetzliche Rahmenbedingungen zu fördern, um eine kontinuierliche Verbesserung der Versorgungsqualität weiterhin zu gewährleisten. Die Rechte der Patienten hinsichtlich der qualitativen Transparenz von Kliniken, Operateuren und Implantatsystemen, letztere unter risikoabhängiger Auswertung müssen gewahrt werden.

## Limitation

- Die Teilnahme am EPRD ist freiwillig. Daher ist die Nachverfolgbarkeit der Patienten in der EPRD-Klinikauswertung auf Versicherte der vdek- und AOK-Kassen beschränkt ist, sodass die Daten von etwa einem Viertel der Patienten unberücksichtigt bleiben.
- Insbesondere fallzahlschwächere Kliniken, die tendenziell schwächere Ergebnisse aufweisen könnten, sind im EPRD unterrepräsentiert. Dies führt dazu, dass der Vergleich, dem sich die teilnehmenden Kliniken im Rahmen des EPRD stellen, möglicherweise strenger ausfällt.
- Die Auswertung bildet nur die gesamte Institution und nicht den einzelnen Operateur ab. Dies ist, insbesondere vor dem Hintergrund hoher Personalfluktuationen, als Limitation zu sehen.

## Ausblick

Die bisherigen Entwicklungen im Bereich der Qualitätssicherung in der Endoprothetik, wie transparente Registerdaten und gezielte Rückmeldesysteme für Kliniken, haben sich als effektive Werkzeuge zur Verbesserung der Versorgungsqualität etabliert. Diese erfolgreichen Ansätze sollten weiter gestärkt und umfassender genutzt werden, um die vorhandenen Potenziale voll auszuschöpfen. Statt neue Regelungen einzuführen, liegt der Fokus darauf, bestehende Systeme wie das EPRD einschließlich des Rückmeldesystem zu optimieren und noch stärker in die klinische Praxis zu integrieren, welches durch das EndoCert-Zertifizierungssystem gefördert und geprüft werden kann. Durch die konsequente Anwendung dieser etablierten Maßnahmen kann eine nachhaltige Qualitätssteigerung in der Endoprothetik erreicht werden, ohne die Beteiligten mit zusätzlichen administrativen Anforderungen zu belasten, die keine effektive Verbesserung erwarten lassen.

## Fazit für die Praxis

- Kliniken können ihre Versorgungsqualität durch Rückmeldungen aus den Klinikauswertungen des Endoprothesenregister Deutschland (EPRD) überprüfen und verbessern, insbesondere bei den Wechselereignissen von Hüft- und Knieendoprothesen.
- Die halbjährlichen EPRD-Klinikauswertungen bieten Kliniken die notwendige Transparenz und Möglichkeit, Verbesserungspotenziale frühzeitig zu identifizieren und gegenzusteuern.
- Es konnte keine Abhängigkeit zwischen der Anzahl der durchgeführten Eingriffe und der Qualitätsverbesserung festgestellt werden, was darauf hinweist, dass kleinere Kliniken ebenso von den Qualitätssicherungsmaßnahmen profitieren.
- Die bereits etablierten Qualitätssicherungssysteme wie das EPRD und EndoCert sollten weiter gestärkt und in den klinischen Alltag integriert werden, um eine nachhaltige Verbesserung der Patientenversorgung weiterhin zu gewährleisten.
- Gesetzliche Initiativen wie das Implantateregistergesetz und die Krankenhausreform sollten die bestehenden Qualitätssicherungssysteme unterstützen, anstatt neue Regelungen einzuführen.

## Data Availability

Die in dieser Studie analysierten Datensätze stehen auf begründete Anfrage hin zur Verfügung. Anfragen können an die korrespondierenden Autoren gestellt werden, vorbehaltlich geltender Datenschutzbestimmungen und ethischer Vorgaben.

## Abkürzungen

DKG: Deutsche Krankenhausgesellschaft
EPRD: Endoprothesenregister Deutschland
EPRD: German Arthroplasty Registry
HTEP: Hüftendoprothesen
IRD: Implantateregister Deutschland
IRegG: Implantateregistergesetz
KTEP: Knieendoprothesen
NJR: Britisches Endoprothesenregister
NRW: Nordrhein-Westfalen
QS: Qualitätssicherung
QSS: Qualitätssicherungssystem
SRR: „Standardised revision ratios“
SWV: Standardisierte Wechselverhältnisse
THA: „Hip arthroplasties“
TKA: „Knee arthroplasties“
